# The association of MOV10 polymorphism and expression levels with preeclampsia in the Chinese Han population

**DOI:** 10.1002/mgg3.1564

**Published:** 2020-12-02

**Authors:** Qian Tang, Ling Wang, Renmei Cai, Lu Zhang, Xiaoxiao Zhang, Xuemei Liu, Shiguo Liu

**Affiliations:** ^1^ Medical Genetic Department the Affiliated Hospital of Qingdao University Qingdao China; ^2^ Prenatal Diagnosis Center the Affiliated Hospital of Qingdao University Qingdao China; ^3^ Department of Nephrology the Affiliated Hospital of Qingdao University Qingdao China; ^4^ Prenatal Diagnosis Center Qingdao Municipal Hospital Qingdao China

**Keywords:** expression, Moloney leukemia virus 10, placenta, polymorphism, preeclampsia

## Abstract

**Background:**

To assess the relationship between *MOV10* rs2932538 polymorphism and susceptibility to preeclampsia (PE) in the Chinese Han population and to investigate whether the placental expression of *MOV10* have association with PE.

**Methods:**

We enrolled 1021 pregnant women with PE and 1594 normotensive pregnant women to analyze genotyping of *MOV10* rs2932538. Clinical data and related test results of all subjects were collected and analyzed. For volunteers providing placentas, real‐time PCR, Western blot, and immunohistochemistry were applied to assess the expression level of *MOV10*.

**Results:**

There was significant statistical difference between preeclamptic patients and healthy subjects in genotype distributions and alleles. The frequencies of genotypes TT+CT were significantly associated with the increased risk of preeclampsia. Besides, T alleles were found to be related to a higher risk of PE. Significant statistical difference was also observed on distributions of genotype in PE without/with severe features group compared or early onset/late onset versus controls. The placental expression of *MOV10* was lower in preeclamptic women, however, no relationship was found between *MOV10* expression level and *MOV10* rs2932538 genotypes.

**Conclusion:**

This study suggests that *MOV10* rs2932538 polymorphism may be associated with PE susceptibility in the Chinese Han population. The placental expression of *MOV10* decrease in PE but have no relationship with rs2932538 polymorphism.

## Graphical abstract

There was relationship between MOV10 polymorphism and preeclampsia. The expression level of MOV10 was lower in preeclamptic placenta. MOV10 expression level shows no relationship with MOV10 rs2932538 genotypes.

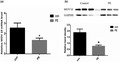

## 1 INTRODUCTION

Preeclampsia (PE) is a peculiar disorder of gestation period characterized by newly occurred hypertension after 20 weeks of gestation or a further increase in blood pressure based on the original hypertension, accompanied by the recent emergence of proteinuria or other multisystem damage (Force et al., 2017). As one of the leading causes of neonatal morbidity and mortality in maternal and fetuses, PE complicates ~3%–5% pregnancies (Mol et al., 2016; Pasha et al., 2017). The World Health Organization assessed that PE brought the death of over 70,000 pregnant women and 500,000 newborns each year (Ushida et al., 2016).

The mechanism of PE has not been completely reveled yet (Berry & Atta, 2016). Previous evidence showed that the pathogenesis of PE involved in dysangiogenesis and endothelial dysfunction (Maynard et al., 2003), ischemia and hypoxia of placenta or trophoblast (Korkes et al., 2017), oxidative stress (Aouache et al., 2013), disturbance of immunoregulation (Laresgoiti‐Servitje 2013), and genetic variation (Lachmeijer et al., 2002), and so on, among which the genetic factors always contribute to the disease susceptibility. However, the relationship of a particular genetic variant and PE is inconsistent due to diversity of regions and ethnicities (Zhan et al., 2015).

Considering PE typically manifested as increased blood pressure, various candidates genes associated with hypertension have been adopted to study the susceptibility of PE. Moloney leukemia virus 10 (*MOV10*, NC_000001.11, OMIM# 610742), first found in the Moloney murine leukemia viruses‐infecting mouse strain, mapped to chromosome 1p13.2 and contained 1003 amino acids (Abudu et al., 2012). It consists of seven putative RNA helicase motifs and is classified as a superfamily 1 (SF1) RNA helicase, playing a key role during the process of RNA interference and assisting host resist to retroviral attack due to its broad antiretroviral activity (Skariah et al., 2017; Wang et al., 2010; Zhang et al., 2016). In addition, MOV10 was discovered to participate in the regulation of blood pressure by influencing the proliferation process of vascular smooth muscle cells (Hong et al., 2013). Based on previously findings, MOV10 can decrease the expression of the tumor suppressor INK4α and inhibit vascular smooth muscle cell proliferation (El Messaoudi‐Aubert et al., 2010; Hong et al., 2013).

A previous Genome‐wide association study (GWAS) of blood pressure using a multistage design in European populations by the International Consortium for Blood Pressure Genome Wide Association Studies have identified the *MOV10* rs2932538 as a locus significantly associated with hypertension (Ehret et al., 2011). Another GWAS of blood pressure and hypertension in Chinese population replicated the loci was associated with blood pressure at genome‐wide significance, SNPs in *CASZ1*, *FGF5*, *CYP17A1*, and *MOV10* were included (Lu et al., 2015). Furthermore, a case‐control study conducted recently in the Chinese Han population proved that polymorphism of rs2932538 in *MOV10* gene have relationship with the increased risk of hypertension (Hong et al., 2013).

In view of all the above mentioned, we hypothesized that *MOV10* might involve in the pathogenesis of PE. Therefore, the aim of this study is to evaluate the association between *MOV10* polymorphism and PE susceptibility in the Chinese Han population. Furthermore, the expression of *MOV10* in the PE placenta was detected to investigate whether the placental expression of *MOV10* have association with PE. Indeed, our study is the first effort to investigate the association between genetic variation of *MOV10* and the onset of PE, and to further validate whether *MOV10* acts as a potential pathogenic gene for PE.

## 2 MATERIALS AND METHODS

### 2.1 Ethical compliance

Our study has obtained approval of the Ethics Committee of the Affiliated Hospital of Qingdao University.

### 2.2 Subjects

We recruited 1021 patients with PE admitted by Affiliated Hospital of Qingdao University, Shandong Provincial Hospital, Liaocheng People's Hospital and another several hospitals as case group from January 2016 to January 2018, meanwhile, 1599 normal pregnant women served as control group. According to the “Guidelines for the diagnosis and treatment of hypertensive disorders of pregnancy (2015)” issued by the Group of Hypertensive Diseases in Pregnancy of Obstetrics and Gynecology Branch of Chinese Medical Association, PE is defined as systolic blood pressure ≥140 mmHg and/or Diastolic blood pressure ≥90 mmHg after 20 weeks of gestation, followed by the excretion of urinary protein ≥0.3 g/24 hr, or urine protein/creatinine ratio ≥0.3, or random urine protein ≥1+ by dipstick.

The control group matched with age of case group but without clinical history of PE, chronic hypertension, heart disease, kidney disorders, diabetes mellitus, hepatic diseases, transfusion, or immunotherapy. In addition, those who experienced artificial insemination, twin/multiple pregnancy should be excluded as well. All subjects in our research were Han Chinese and informed consent was signed before participating in our trial.

### 2.3 Genotyping

After peripheral venous blood collected were anticoagulated with EDTA, genomic DNA was extracted from 200 μl whole blood using TIANamp Blood DNA kit (TIANGEN). Real‐time PCR was conducted to amplify relevant DNA fragments using TaqMan probe designed and synthesize by (Thermo Fisher Scientific, CN). The sequence of the forward of *MOV10* rs2932538 was 5ʹ‐TTCATTCTGCGGGAAAAAAATAGAT‐3ʹ and the reverse primer was 5ʹ‐GTGGGGCTACAATAACAAACCTTTG‐3ʹ. The polymerase chain reaction (PCR) contained 1.25 μl 20× probe and primers, 12.5 μl 2 × PCR Genotyping Master Mix, and 11.25 μl DNA and DNase‐free water formed 25 μl reaction mixture. The amplifications were carried out with the following protocol: 95°C for 3 min, 45 cycles at 95°C for 15 s, and 60°C for 1 min. For each cycle, the fluorescent signal from the VIC‐ or FAM‐labeled probe was determined. This SNP was genotyped using TIB‐8600 Real‐Time Quantitative Thermal Cycler (Triplex International Biosciences Co., Ltd.). The genotypes were distinguished according to the strength of the fluorescent signals from VIC/FAM‐labeled probes.

### 2.4 Placental tissue

The central area (~2 cm^2^) of placental tissue was obtained immediately after delivery from the preeclamptic and control groups. After washing of the maternal blood with physiological saline, tissues were immediately frozen in liquid nitrogen and stored. The rest of the fresh placental tissues were embedded in paraffin and cut to slices for immunohistochemistry. Total RNA was extracted from placenta using RNAiso Plus (Takara), and transcribed into cDNA by PrimeScript reagent Kit (Vazyme Biotech Co., Ltd). The protein was extracted using RIPA Lysis Buffer (Beyotime) and PMSF (Solarbio). BCA Protein Assay kit (CWBIO) was used to quantify the concentration of protein by Microplate Photometer (Thermo Fisher Scientific).

### 2.5 Real‐time PCR

The 20 μl reaction mixture contained 0.4 μl forward primer, 0.4 μl reverse primer, 10 μl SYBR qPCR Master Mix, cDNA, and ddH_2_O. GAPDH was used as an internal control. The primers for *MOV10* were: forward: 5ʹ‐GTGCTGATGTCGTGGATCGA‐3ʹ and reverse: 5ʹ‐TCACTGTGGCAGCCTCTTCA‐3ʹ. The primers for GAPDH were forward: 5′‐CATGTTCGTCATGGGTGTGAA‐3′ and reverse: 5′‐GGCATGGACTGTGGTCATGAG‐3′. Amplification reaction under the following conditions: 95°C for 30 s for 1 cycle; 95°C for 10 s and 60°C for 30 s for 40 cycles, and fluorescence was quantified.

### 2.6 Western blot

The *MOV10* protein and internal reference were separated by SDS–PAGE in 10% Triseglycine gels. Polyvinylidene fluoride (PVDF) films were transferred in cold blocking buffer. Blocking the membrane in 5% notfat milk dissolved in 1x TBST for 1 hr at room temperature. The films with carrying separated protein were incubated with *MOV10* rabbit anti‐human antibodies (1:600) and GAPDH rabbit anti‐rabbit antibodies (1:10000) for, respectively, overnight at 4℃. After washing, the membranes were incubated with Horseradish peroxidase (HRP)‐conjugated secondary antibodies (1:5000) for 1 hr. The membranes were placed into the chemiluminescent reagent to get full exposure, and the HRP signal was captured by Light‐Capture with a cooled CCD camera (UVPs). Signals were quantified using ImageJ software.

### 2.7 Immunohistochemistry

The 5 μm placental tissue sections were dried and deparaffinized. Then, sections were soaked in EDTA Antigen Retrieval Solution (BOSTER) and heated at 95℃ for 15 min. Subsequently, 3% H_2_O_2_ solution was dropped on the sections to inactivate endogenous enzymes for 10 min. The 5% goat serum configured in 1×PBS was added to sections and maintained for 30 min at room temperature. Sections reacted with primary antibody (10370‐1‐AP, Rabbit, 1:200, Proteintech) and negative with rabbit IgG for 1 hr at 37℃, and polymerized HRP‐labeled anti‐rabbit IgG (BOSTER) was dropped to incubate at 37℃ for 30 min. DAB reagent kit (BOSTER) was used to carry out color reaction for 8 min at room temperature. The sections were observed under the microscope after another dehydration and resin enveloping.

### 2.8 Statistical analysis

Hardy–Weinberg equilibrium was analyzed to affirm the characters of group representation. We used Student's *t*‐test to compare the clinical data and relative mRNA expression level between cases and controls. Cochran–Armitage test was performed to compare the genotypic distributions of different groups. Genetic model and allele frequencies were assessed using chi‐square test. The odds ratio (OR) and the 95% confidence interval (95% CI) were calculated for *MOV10* SNP. Otherwise, one‐way ANOVA followed by Bonferroni‐corrected for multiple comparisons was conducted to analyze the relationship between genotype and the relative *MOV10* mRNA expression. The *p* < 0.05 was considered statistically significant. All data analysis involved was performed with SPSS.22 (IBM).

## 3 RESULTS

### 3.1 Demographic and clinical characteristics

The clinical characteristics and biochemical features of the subjects recruited were presented in Table 1. The maternal age of two groups matched (*p* = 0.094). The gestational age of PE patients was significantly lower (*p* < 0.001) compared to healthy group. The pregestational body mass index (PBMI) and gestational weight gain of preeclamptic women were significantly higher than normal subjects (*p* = 0.021; *p* < 0.001). In addition, the case group had higher blood pressure (*p* < 0.001) but lower neonatal weight (*p* < 0.001). In terms of laboratory markers, the levels of ALT, AST, blood urea nitrogen, serum creatinine, and blood uric acid of the case group were significantly higher than the control group (Table 1).

**TABLE 1:** Clinical and biochemical features of cases and controls.

Features	Cases (n = 1017)	Controls (n = 1594)	*t*‐value	*p*‐value
Maternal age (years)	31.9 ± 5.4	31.5 ± 4.4	1.678	0.094
Gestational age (weeks)	35.3 ± 3.3	39.3 ± 1.2	−33.351	<0.001
Pregestational body mass index (PBMI; k/m^2^)	23.8 ± 3.1	23.4 ± 3.0	2.303	0.021
Gestational weight gain (kg)	17.7 ± 7.0	15.6 ± 4.4	6.11693	<0.001
Neonatal weight (g)	2426.5 ± 846.8	3400.5 ± 337.7	−27.198	<0.001
Systolic pressure (mmHg)	163.1 ± 18.1	117.1 ± 10.2	71.27	<0.001
Diastolic pressure (mmHg)	105.8 ± 12.8	74.8 ± 7.1	67.959	<0.001
ALT (U/L)	22.3 ± 19.4	12.9 ± 8.0	13.190	<0.001
AST (U/L)	27.2 ± 17.4	18.0 ± 7.1	14.542	<0.001
Blood urea nitrogen (mmol/L)	4.8 ± 1.9	3.2 ± 0.9	21.519	<0.001
Serum creatinine (μmol/L)	59.6 ± 15.8	51.0 ± 13.3	11.959	<0.001
Blood uric acid (μmol/L)	386.0 ± 89.1	271.5 ± 70.4	28.269	<0.001

Note

Data are shown as the mean ±standard deviation.

### 3.2 Genetic analysis

The genotypic distribution of this candidate SNP in the case group (*χ* ^2^ = 0.202, *p* > 0.05) and control group (*χ* ^2^ = 2.360, *p* > 0.05) conformed to Hardy–Weinberg equilibrium. The frequencies of genotype and alleles for *MOV10* rs2932538 polymorphism in cases and controls were shown as Table 2. In our study, the analysis of the genotypic distributions of rs2932538 in *MOV10* revealed the significant differences between preeclamptic patients and healthy subjects (*χ* ^2^ = 26.107, *p* < 0.001). The frequencies of the genotypes TT+CT were significantly associated with the risk of PE rather than genotype CC (*χ* ^2^ = 27.938, *p* < 0.001, OR = 1.605, 95% CI = 1.346–1.915) in the dominant model. The analysis of alleles showed that T allele carriers had a higher risk of PE when compared to C allele carriers (*χ* ^2^ = 28.061, *p* < 0.001, OR = 1.517, 95% CI = 1.299–1.771).

**TABLE 2:** Genotype and allele frequencies of *MOV10* rs2932538 in cases and controls.

	Cases n (%)	Controls n (%)	*χ* ^2^	*p*‐value
Genotypes				
CC	691 (67.9)	1232 (77.3)	26.107	<0.001
TT	35 (3.4)	35 (2.2)		
CT	291 (28.6)	327 (20.5)		
Dominant				
CC	691 (67.9)	1232 (77.3)	27.938	<0.001
TT+CT	326 (32.1)	362 (22.7)		
Alleles				
C	1673 (82.3)	2791 (87.5)	28.061	<0.001
T	361 (17.7)	397 (12.5)		

To further investigate the relationship between *MOV10* polymorphism and PE, we divided patients into groups of PE without severe features and PE with severe features in accordance with the guidelines given by the American College of Obstetricians and Gynecologists. Frequencies of genotype and allele of rs2932538 in PE without/with severe features were compared with healthy pregnant women, respectively. As shown in Table 3, significant statistical differences on genotype distribution and allelic frequencies were found between PE without severe features group and control group (*χ* ^2^ = 3.984, *p* = 0.046 by genotype; *χ* ^2^ = 4.237, *p* = 0.04, OR = 1.395, 95% CI = 1.015–1.918 by allele). Moreover, significant statistical difference was also observed between PE with severe features group and control group both on the genotypic and allelic frequencies of rs2932538 (*χ* ^2^ = 26.418, *p* < 0.001 by genotype; *χ* ^2^ = 27.576, *p* < 0.001, OR = 1.539, 95% CI = 1.309–1.809).

**TABLE 3:** The comparison of genotypes and allele frequencies of rs2932538 between controls and mild/severe PE.

	Genotypes			*χ* ^2^	*p*‐value	Alleles		*χ* ^2^	*p*‐value	OR (95% CI)
	CC n (%)	CT n (%)	TT n (%)			C	T			
Control	1232 (77.3)	327 (20.5)	35 (2.2)	3.984	0.046	2791 (87.5)	397 (12.5)	4.237	0.040	1.395 (1.015–1.918)
PE without severe features	109 (70.8)	39 (25.3)	6 (3.9)			257 (83.4)	51 (16.6)			
Control	1232 (77.3)	327 (20.5)	35 (2.2)	26.418	<0.001	2791 (87.5)	397 (12.5)	27.576	<0.001	1.539 (1.309–1.809)
PE with severe features	582 (67.4)	252 (29.2)	29 (3.4)			1416 (82.0)	310 (18.0)			

Table 4 presents the distribution of genotypes and alleles of rs2932538 between control group and early onset/late onset PE group. There was significant statistical difference on genotypic frequencies of this polymorphism between early onset PE /late onset PE patients and controls (early onset PE *χ* ^2^ = 19.404, *p* < 0.001; late onset PE *χ* ^2^ = 17.086, *p* < 0.001). Furthermore, the frequencies of T alleles were statistical higher in early onset/late onset PE than in the controls (early onset PE *χ* ^2^ = 20.580, *p* < 0.001, OR = 1.521, 95% CI = 1.268–1.825; late onset PE *χ* ^2^ = 17.780, *p* < 0.001, OR = 1.548, 95% CI = 1.262–1.898).

**TABLE 4:** The comparison of genotypes and allele frequencies of rs2932538 between controls and early onset/late onset PE.

	Genotypes			*χ* ^2^	*p*‐value	Alleles		χ^2^	*dP*‐value	OR (95% CI)
	CC n (%)	CT n (%)	TT n (%)			C	T			
Control	1232 (77.3)	327 (20.5)	35 (2.2)	19.404	<0.001	2791 (87.5)	397 (12.5)	20.580	<0.001	1.521 (1.268–1.825)
Early onset PE	405 (68.3)	165 (27.8)	23 (3.9)			975 (82.2)	211 (17.8)			
Control	1232 (77.3)	327 (20.5)	35 (2.2)	17.086	<0.001	2791 (87.5)	397 (12.5)	17.780	<0.001	1.548 (1.262–1.898)
Late onset PE	283 (66.7)	129 (30.4)	12 (2.8)			695 (82.0)	153 (18.0)			

### 3.3 Genotype–phenotype analysis

In order to evaluate the associations of clinical and biochemical features and genotypes of rs2932538 in PE patients, preeclamptic patients were split into three groups based on genotypes. As the results in Table 5, there were no significant differences among the three groups of parameters in demographic characteristics and clinical features. However, the genotype CC group had higher gestational weight gain but lower serum total cholesterol than the genotype CT group (gestational weight gain *p* = 0.031 and total cholesterol *p* = 0.048). In addition, patients were further subdivided into CC+CT/TT group and CC/CT+TT group, however, no statistical difference was observed.

**TABLE 5:** Associations of clinical and biochemical features and genotypes of rs2932538 in PE patients.

Features	(1) CC (n = 691)	(2) CT (n = 291)	(3) TT (n = 291)	(1) versus (2) versus (3) *P*‐value	(1) versus (2) *P*‐value	(1) versus (3) *P*‐value	(2) versus (3) *P*‐value	(1)+(2) versus. (3) *P*‐value	(1) versus. (2) +(3) *P*‐value
Maternal age (y)	31.86 ± 5.39	31.93 ± 5.45	30.72 ± 5.39	0.483	0.864	0.244	0.231	0.232	0.876
Gestational age (weeks)	35.31 ± 3.35	35.27 ± 3.31	35.60 ± 2.94	0.896	0.893	0.668	0.640	0.654	0.988
Pregestational body mass index (PBMI; k/m^2^)	23.76 ± 2.78	23.85 ± 2.64	24.82 ± 3.45	0.401	0.768	0.179	0.237	0.187	0.515
Gestational weight gain (kg)	17.98 ± 6.50	16.62 ± 6.38	18.78 ± 6.08	0.072	0.031	0.610	0.181	0.442	0.062
Systolic pressure (mmHg)	163.38 ± 18.02	163.27 ± 18.16	165.78 ± 17.29	0.752	0.929	0.463	0.456	0.453	0.906
Diastolic pressure (mmHg)	106.00 ± 12.98	105.91 ± 12.25	105.41 ± 12.68	0.966	0.924	0.78	0.832	0.805	0.874
Neonatal weight (g)	2389.15 ± 873.54	2506.46 ± 820.31	2228.17 ± 950.20	0.208	0.134	0.438	0.193	0.344	0.230
Platelets (×10^9^/L)	210.34 ± 66.43	208.74 ± 71.28	230.87 ± 80.31	0.231	0.750	0.103	0.088	0.093	0.881
ALT (U/L)	22.59 ± 19.90	20.95 ± 15.33	26.42 ± 21.97	0.232	0.246	0.269	0.127	0.209	0.442
AST (U/L)	27.72 ± 17.87	26.12 ± 16.31	27.96 ± 17.06	0.456	0.218	0.941	0.578	0.821	0.262
Serum creatinine (μmol/L)	60.47 ± 17.71	59.42 ± 21.50	57.54 ± 15.01	0.570	0.462	0.398	0.600	0.445	0.357
Blood uric acid (μmol/L)	382.87 ± 104.89	385.17 ± 125.56	374.08 ± 92.65	0.887	0.685	0.658	0.645	0.651	0.892
Triglycerides (mmol/L)	3.85 ± 1.46	3.99 ± 1.66	4.23 ± 1.59	0.329	0.303	0.231	0.466	0.280	0.193
Total cholesterol (mmol/L)	6.81 ± 1.89	7.14 ± 1.91	6.85 ± 1.43	0.141	0.048	0.920	0.498	0.895	0.063

Note

Data are shown as the mean ±standard deviation.

### 3.4 The relative mRNA expression level of *MOV10*

**FIGURE 1:**
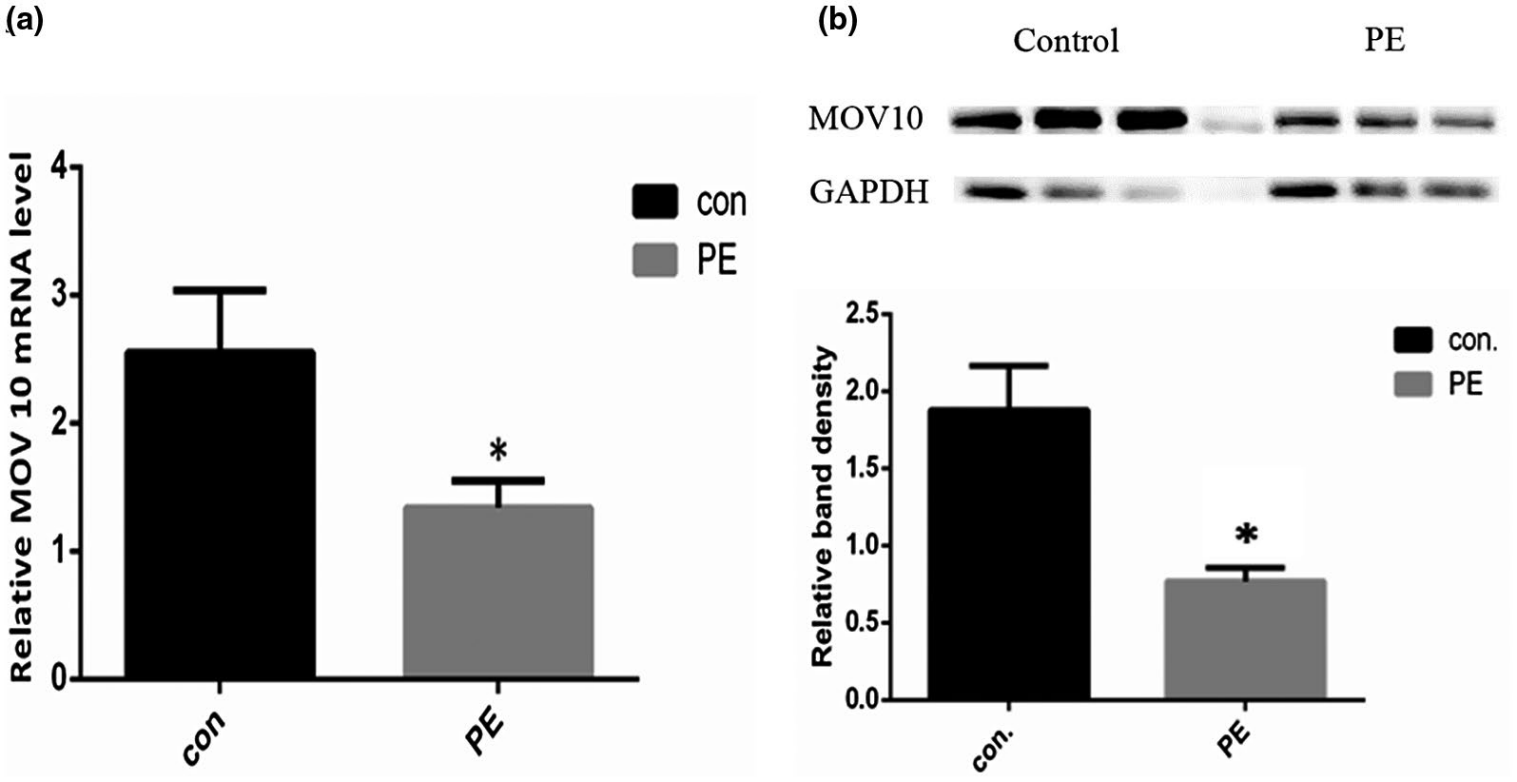
Expression of *MOV10* in placenta. a, Relative mRNA expression level of *MOV10* in placental tissue measured by qPCR. Data for controls (n = 52) and cases (n = 48) were compared. Data represent mean ±SE. *represents *p* < 0.05 versus the control group. b, The expression levels of MOV10 protein in placenta measured by western blot. The relative band density of two groups was processed by ImageJ software. Data represent mean ±SE. *represents *p* < 0.05 versus the control group.

The expression levels of *MOV10* mRNA were measured in placental tissue from 100 subjects containing 52 controls and 48 cases. We found that the level of *MOV10* mRNA was around twofold higher in normotensive pregnant women compared to women PE with significant difference (*p* < 0.05, 2.551 ± 0.488 vs. 1.337 ± 0.212; Figure 1a).

**FIGURE 2:**
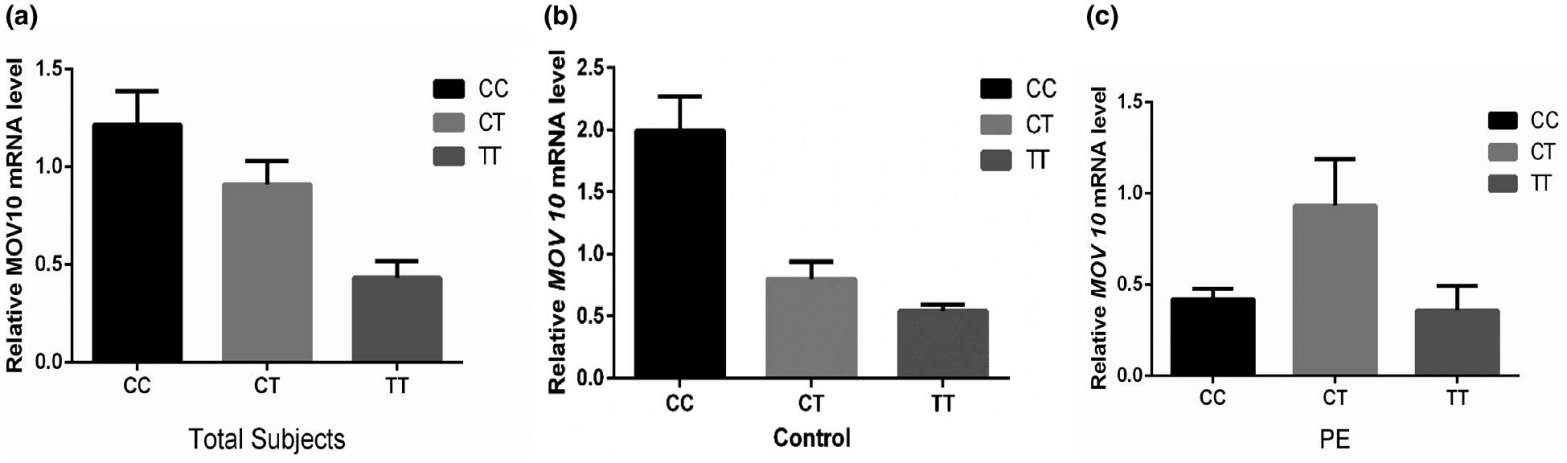
Relative mRNA expression level of *MOV10* in the placental tissue. a, Data for CC (n = 96), CT (n = 23), and TT (n = 5) genotypes in total subjects were compared. b, Data for CC (n = 51), CT (n = 10), and TT (n = 2) genotypes in control group. c, Data for CC (n = 45), CT (n = 13), and TT (n = 3) genotypes in PE group. Data were expressed as mean ±SE.

Next, we tested the *MOV10* mRNA levels based on genotype to investigate whether rs2932538 affects the placental expression of *MOV10*. However, no significant difference was observed among three genotypes in all pregnant women (*p* > 0.05; Figure 2a). Although the relative mRNA expression level of rs2932538 CC genotypes were higher than CT and TT genotypes, the differences were marginally insignificant. We further analyzed the relative *MOV10* mRNA expression level based on genotype in control group and PE group, respectively, and found that the expression level of *MOV10* in control group is higher in CC genotypes than CT and TT genotypes, however, the difference had no statistical significance (*p* > 0.05; Figure 2b). Besides, no difference was observed in *MOV10* mRNA level among three genotypes of CC, CT, and TT in PE group though CT genotypes had higher MOV10 expression level than CC and TT genotypes (Figure 2c).

### 3.5 The protein expression of gene *MOV10* in placenta

We further validated the expression of *MOV10* protein between PE and the normotensive pregnant women by western blot (Figure 1b). The result showed a lower expression of *MOV10* protein in cases comparing with controls (*p* < 0.05), which is consistent with the transcriptional expression of *MOV10* in placentas. In addition, we detected the relative expression of *MOV10* based on genotype in total subjects, control group, and PE group, respectively, but no significant difference was observed among genotypes in these groups (data not shown).

**FIGURE 3:**
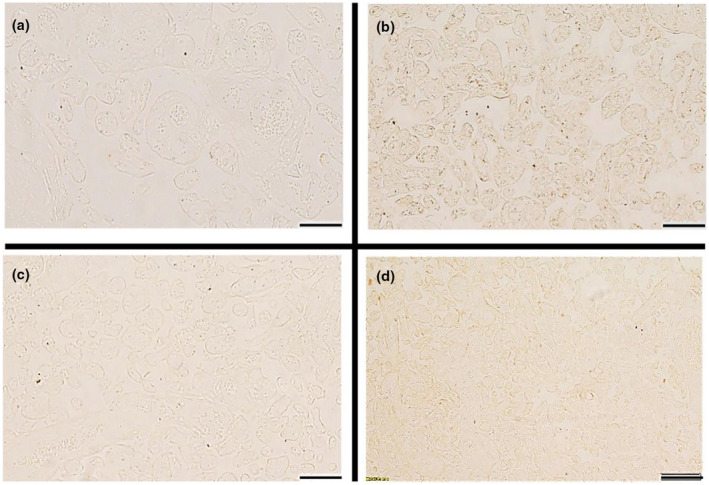
Immunohistochemical staining of MOV10 in the placental tissue. a, Normal group with rabbit IgG (as negative control). b, Normal group with MOV10 antibody. c, Preeclampsia group with rabbit IgG (as negative control). d, Preeclampsia group with MOV10 antibody. Scale bars, 200 μm.

In addition, immunohistochemical analysis of *MOV10* protein in placenta tissue was performed. Positive signals were detected in placental chorionic syncytiotrophoblast cells. The staining of cells was lighter and the detected signal was weaker in the case group, while controls had a darker signal, suggesting that *MOV10* protein was expressed in syncytiotrophoblast, and PE had a lower expression level (Figure 3).

## 4 DISCUSSION

PE is defined as a pregnancy‐specific disease resulting from vascular dysfunction and abnormity of hemodynamics that multi‐system involved including the liver, kidney injury, hematologic abnormalities, and metabolic disturbance, and so on (Berry & Atta, 2016; Muller‐Deile & Schiffer, 2014). Our statistical data shows that patients with PE have higher ALT, AST, blood urea nitrogen, serum creatinine, and blood uric acid than normal pregnancy women, providing an explication for multiple organ dysfunction caused by PE. Smaller gestational age, lighter neonatal weight but higher PBMI, and gestational weight gain were observed in PE women, which is consistent with previous studies concerning the risks of PE (Eiland et al., 2012; Galaviz‐Hernandez et al., 2016).

Although the pathogenesis of PE is not yet clear, it is generally believed that PE originates from placenta and relieves the symptoms after the delivery of placenta (Iriyama et al., 2015; Roberts & Escudero, 2012). In normal pregnancy, trophoblast cells infiltrate into the muscular layer of uterine spiral arteries and decidua, leading to the reconstruction of spiral arteries, dilatation of lumen, reduction of blood flow impedance, and the augment of blood flow (Harati‐Sadegh et al., 2018). While in PE, the incomplete spiral artery remodeling results in the incomplete transformation of uterine spiral arteries, reducing uterine blood flow, and fetal blood supply (Goulopoulou, 2017). Researches shows that the incomplete remodeling of the uterine spiral artery results in vasospasm and placental ischemia, and then, systemic mediators are released from ischemic placenta tissue into the maternal circulation, leading to inflammation and vascular endothelial damage, which causes a series of symptoms of PE (Roberts & Hubel, 2018). Considering the key role of placenta in the development of PE, the polymorphism of genetic factors in PE placenta may affect a lot on pathogenesis of PE (Harati‐Sadegh et al., 2018).

Genetic variations of multiple candidate genes for hypertension were often found to be significantly correlated with susceptibility to PE (Johnson et al., 2009; Kvehaugen et al., 2013; Serrano et al., 2004; Shim et al., 2007; Wang et al., 2018; Xia et al., 2012). Hong et al., (2013) indicated that the genetic variant of *MOV10* was related to hypertension susceptibility, which was consistent with the previous results of GWASs of hypertension (Lu et al., 2015; Ehret et al., 2011).

The rs2932538, located in noncoding region of *MOV10*, was proved to have association with hypertension in Europeans and Chinese (Ehret et al., 2011; Hong et al., 2013; Lu et al., 2015). Our study indicated that polymorphism of rs2932538 in *MOV10* gene was associated with susceptibility to PE as a result of significant differences between patients of PE and controls on genotype and allele frequencies. Besides, pregnant women with genotypes TT+CT were at higher risk of PE rather than those with genotype CC, which coincided with the previous study of *MOV10* conducted in patients with hypertension in the Chinese Han population (Hong et al., 2013). The difference of alleles distribution between preeclamptic pregnant women and normal pregnant women suggests that the individuals carrying T allele may have an increased susceptibility to PE than C allele carriers.

In addition, the genotypic distributions allelic frequencies showed statistical significance between normotensive women and mild/severe PE, suggesting that *MOV10* rs2932538 polymorphism may not affect the severity level of PE. Furthermore, the genotypic distributions and frequencies of allele in early onset/late onset PE were also found to have association with this polymorphism in our study. Stated thus, it is likely that SNP rs2932538 in *MOV10* is connected with susceptibility to PE.

One important thing to note is that a risk allele of rs2932538 for PE was T allele based our findings, which is inconsistent with a previously reported paper that a risk allele for hypertension was C allele in Han Chinese population. The reason may be the existence of discrepancy on the relationship between SNP and related disease in different areas, while the sufficient subjects recruited in our research are aimed at pregnant women with PE and mainly concentrated in northern China. Alternatively, replication study could be carried out to validate the mentioned experimental results.

It is well known that the origin of PE is the placenta and PE symptoms diminishes after delivery or placentas removal (Roberts & Escudero, 2012). Altered expression of several genes in the placenta of pregnancy complicated with PE has been described, which may contribute to pathogenesis of this condition (Eskandari et al., 2018). In our research, we found lower expression of *MOV10* in placenta of preeclamptic women, implying that *MOV10* may be associated with the PE. Further study are needed to determine that whether the downregulation of *MOV10* is the result of PE or a reason for disease development.

SNP located in intron region may have an impact on gene function by effecting gene transcription level, gene splicing, and other mechanisms (Sturm et al., Feb, 2008). However, we did not find association between rs2932538 polymorphism and the expression level of *MOV10*. No significant difference of *MOV10* expression levels were observed among three genotypes in total subjects, healthy pregnant women, and preeclamptic patients, respectively. Of course, the fact that few quantity samples may cause experimental error, especially the lack of uncommon genotype TT, so it demands enlargement of sample size.

Actually, our study has several limitations. First of all, participants are mainly Northern Han Chinese Population, so regional, and ethnic difference cannot be ignored. The findings need to be replicated that larger samples and more extensive genetic studies are involved. Moreover, our experiments mainly involved in the expression of *MOV10* gene and protein, no specific mechanism, and functional study were mentioned. Therefore, further study will be committed to continue on the pathogenesis of *MOV10* on PE. In a word, our study indicated that genetic variation of *MOV10* is in relationship with PE susceptibility, contributing to find potential diagnosis and therapeutic targets for PE, which will provide new an idea for the prevention and treatment of PE.

## 5 CONCLUSION

In conclusion, we first found that polymorphism of rs2932538 in *MOV10* was associated with susceptibility to PE by detecting genotypes and alleles. Furthermore, our results showed that the low placental expression of *MOV10* may be associated with PE. In addition, we also found that the genetic variation of this locus was not associated with the gene expression level in the scope of this study.

## DISCLOSURES

The Authors declare that there is no conflict of interest.

## ETHICAL APPROVAL

All procedures performed in studies involving human participants were in accordance with the ethical standards of the Ethics Committee of the Affiliated Hospital of Qingdao University and written consent was obtained from all of the pregnancies before the collection of the placenta and blood.
